# Inhibition of Rho/ROCK signaling pathway participates in the cardiac protection of exercise training in spontaneously hypertensive rats

**DOI:** 10.1038/s41598-022-22191-3

**Published:** 2022-10-25

**Authors:** Mengwei Li, Limei Zhang, Xinyan Liu, Guoqiang Wang, Jian Lu, Jifeng Guo, Hongjie Wang, Jinpeng Xu, Yi Zhang, Na Li, You Zhou

**Affiliations:** 1 Department of Physiology, School of Basic Medical Sciences, Hebei University, 342 Yu Hu Dong Rd., Baoding, 071000 People’s Republic of China; 2 Clinical School of Medicine, Hebei University, Baoding, 071000 People’s Republic of China; 3 Hengshui People’s Hospital, Hengshui, 053000 People’s Republic of China; 4 Department of Physiology, School of Basic Medical Sciences, Hebei Medical University, Shijiazhuang, 05000 People’s Republic of China; 5 Hebei Provincial Key Laboratory of Skeletal Metabolic Physiology of Chronic Kidney Disease, Baoding, 071000 People’s Republic of China

**Keywords:** Cardiovascular biology, Neurophysiology

## Abstract

Exercise training (ExT) is capable of improving the heart function of spontaneously hypertensive rats (SHRs), but the underlying molecular mechanisms remain elusive. This study was aimed to investigate whether inhibition of RhoA/ROCK signaling pathway contributes to the cardiac protection by low-intensity ExT in SHRs. The results demonstrated that, compared with Wistar-Kyoto (WKY) rats, SHRs obviously exhibited higher blood pressure, increased heart weight index and thickness of left ventricular wall, decreased left ventricular function, damaged myocardial construction, and increased collagen fiber of left ventricle (P < 0.05 or P < 0.01). Meanwhile, the mRNA and protein expression levels of RhoA and ROCK in the heart of SHRs were significantly increased, compared with those of WKY rats (P < 0.05 or P < 0.01). Interestingly, the pathological changes of heart aforementioned were all improved in SHR-ExT rats compared with SHR-Sed rats (P < 0.05 or P < 0.01), indicating the cardiac protection of exercise training. In addition, the cardiac protective effect of exercise training could be blocked by LPA, an activator of Rho/ROCK signaling, and the protective effect in SHR rats could be mimicked by Fasudil, an inhibitor of Rho/ROCK signaling. The results strongly suggest that low-intensity ExT can protect heart against structure and function through inhibiting Rho/ROCK signaling pathway in hypertensive rats.

## Introduction

Hypertension is a common global disease caused by genetic and environmental factors, which is a risk factor for cardiovascular disease and seriously endangers human health^1^. Hypertension, coronary heart disease (CHD) and dilated cardiomyopathy (DCM) are the main causes of heart failure. Chronic or persistent hypertension can cause adverse myocardial remodeling by inhibiting or activating several signaling pathways in myocardial tissue, and even lead to sudden cardiac death^2^. Although the common anti-hypertensive drugs can regulate the blood pressure level to a certain extent, but they can’t prevent the occurrence of malignant cardiovascular events^3^. The side effects of drugs cannot be ignored, so it is urgent to explore a safe and effective way to prevent or reverse hypertension and its complications.

As we all know, exercise is benefit for physical health, especially plays a positive role in the prevention and treatment of hypertension, without side effects caused by drugs. Previous studies have shown that the lifestyle such as exercise training (ExT), low salt diet and weight control can effectively reduce the morbidity and mortality of cardiovascular diseases, ameliorate the prognosis of patients, and improve the quality of life^4^. As an optimal exercise prescription, low- and moderate-intensity ExT have been proved to produce anti-hypertensive effect on patients with hypertension^5,6^. A recent report demonstrated that ExT can significantly reduce blood pressure, improve left ventricular function and alleviate myocardial fibrosis in SHRs^7^. However, the regulatory mechanisms underlying cardiovascular protection of ExT are still not clear.

ROCK, known as a serine/threonine kinase, is the downstream signal molecule of RhoA protein. RhoA and ROCK proteins of the RhoA/ROCK signal pathway in multiple tissues (such as nervous system, heart and uterus) exhibit an abnormal high expression level and activity in patients and SHRs^8^. Activation of Rho/ROCK pathway can cause phosphorylation of its substrate MLCP, subsequently lead to MLCP inactivation and muscle contraction. As a non-Ca^2+^ dependent signaling pathway, RhoA/ROCK activation can also increase the Ca^2+^ sensitivity of smooth muscle cells through inhibiting myosin activity, thus leading to coronary spasm and accelerating the process of myocardial damage^9^. Additionally, activation of ROCK1 up-regulates TGFβ1 to activate cardiac fibroblasts and increase fibrosis of cardiomyocytes^10^. Rho-kinase inhibitor can relieve adverse cardiac remodeling after myocardial infarction. The down-regulation of ROCK can reduce cardiac fibrosis^11^, and specific deletion of ROCK2 in fibroblasts can alleviate cardiac hypertrophy, fibrosis and diastolic dysfunction through inhibiting the production of fibroblast growth factor (FGF)^12^. Also, destruction of ROCK1 and ROCK2 in cardiomyocytes can inhibit cardiac fibrosis remodeling by promoting autophagy^13^. The data clearly suggest that Rho/ROCK signal pathway is closely involved in the pathological myocardial remodeling. A recent study showed that ROCK maybe a cardiac hypertrophy modulator in obesity and physical exercise^14^. Therefore, we hypothesize that low- and moderate- intensity ExT can protect heart through inhibition of Rho/ROCK signal pathway. The present study aims to determine the protective effect of ExT on cardiac remodeling and function, and whether is RhoA/ROCK signaling involved.

## Materials and methods

### Experimental animals

8-week-old male spontaneously hypertensive rats (SHRs) and Wistar-Kyoto (WKY) rats (weight 200–230 g) were purchased from Beijing Vital River Laboratory Animal Technology Co., Ltd. (Beijing, China) and maintained on a 12 h dark/light cycle in a specific pathogen-free facility, in which the room temperature was 22–25 ℃, and the humidity was 40–70%. Water and standard chow were provided ad libitum. The experimental protocols were approved by the Animal Care and Use Committee of Hebei University and were conducted in accordance with the Guidelines for Care and Use of Laboratory Animals issued by Hebei University (No. IACUC-2019002SR). All efforts were made to minimize the number of animals used and their suffering. All experiments were carried out in compliance with the ARRIVE guidelines.

### Exercise training program

**Figure 1:**
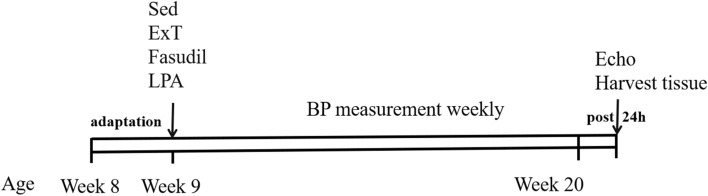
Scheme of exercise training and drug treatment.

After the adaptive feeding for one week, all WKY and SHR rats (9-week-old) were randomly divided into six groups (6–8 rats in each group): SHR control (SHR-Sed), WKY control (WKY-Sed), SHR plus exercise training (SHR-ExT), WKY plus exercise training (WKY-ExT), SHR exercise training plus LPA (Lysophosphatidic acid) (SHR-ExT-LPA) and SHR plus Fasudil (SHR-Fasudil). The rats experiencing exercise training were given low intensity swimming (60 min per day, 5 days per week, 12 weeks) in middle-sized rat water maze (Shenzhen Rayward Life Technology Co., LTD, China). In the meantime, the SHR-ExT-LPA and SHR-Fasudil rats were intraperitoneally injected with 1 mg/kg LPA (Avanti Systems, USA) and 15 mg/kg Fasudil (Qingdao Jinfeng Pharmaceutical CO., LTD, China), respectively (Fig. 1). LPA, known as ROCK agonist was used to verify the role of Rho/ROCK signaling pathway in ExT-induced anti-hypertension. Fasudil, a potent ROCK inhibitor widely applied in clinical anti-hypertension was used to test whether there is similar anti-hypertension between ExT and Fasudil. Systolic blood pressure (SBP) was measured consciously using non-invasive blood pressure system (BP-600A, Techman Instrument, China) every weekend. All measurements were taken 24 h after termination of the ExT session in order to eliminate the stress affection induced by ExT.

### Echocardiography measurement

Twenty-four hours after the last ExT session, the rats were anesthetized with isoflurane, and chest hair was removed using hair remover cream. The heart function and left ventricular wall thickness were assessed by an echocardiography (VisualSonics, Inc., Toronto, Canada). Ejection fraction (EF), fractional shortening score (FS), stroke volume (SV), left ventricular end-systolic dimension (LVESd), left ventricular end-diastolic dimension (LVEDd), the thickness of the left ventricular posterior wall in diastole; diastolic (LVPWd), the thickness of left ventricular posterior wall during systole; systolic (LVPWs), systolic interventricular septal (IVSs), diastolic interventricular septal (IVSd), relative wall thickness (RWT), left ventricular mass index (LVMI) were determined according to M-mode tracings by Vevo2100 ultra high resolution ultrasound imaging system for small animals.

### Histological analysis of LV

After echocardiography, the rats were deeply anesthetized with 2% pentobarbital sodium (50 mg/kg), the heart was quickly removed. The fat and connective tissue around the heart were removed, the blood was washed out in 4 °C saline. The picture of heart was taken with a camera (Fujifilm, Japan).

### Hematoxylin–eosin (HE) staining

Rats were sacrificed under deep anesthesia, aortic retrograde perfusion was performed with cold phosphate-buffered saline (PBS, pH 7.3) and 4% polyformaldehyde. Then, the hearts were excised and post-fixed with polyformaldehyde for 6 h at room temperature, dehydrated with 30% ethanol, embedded in paraffin blocks, and cross-sectioned into 4 μm thick slices. The slices were roasted for 3 h, dewaxed by xylene and gradient ethanol. The dewaxed slices were placed in Harris hematoxylin solution for 5 min to stain the nuclei, and washed with tap water for 3 times, about 30 s each time. The slices were washed again with 0.1% hydrochloric acid ethanol for 1 s, then washed to remove the excess dye, and stained with 0.5% eosin alcohol solution for 2 min to color the cytoplasm, then dehydrated by ethanol, xylene transparent slices and neutral gum sealed slices. The diameter of cardiomyocytes with visible intact cellular membranes was measured. The widths of the individual cardiomyocyte were manually measured across the middle of the cellular membranes with Image J software. The average diameter of rat cardiomyocyte was calculated from approximately 60 cardiomyocytes. Those who were responsible for data collection were blind to the grouping.

### Masson dyeing

The above dewaxed and rehydrated sections were put into a wet box, and an appropriate amount of Weigert ferrioxylamine A and B were mixed in equal amounts. The sections were stained with acidic ethanol differentiation solution for 5–10 min, with ammonia blue return for several seconds, with the fuchsin staining solution for 5–10 min, and washed with acetic acid working solution for 1 min. Then the sections were treated with phosphomolybdenum solution for 1–3 min, and Aniline blue solution for 1–2 min. The sections were dehydrated rapidly with 95% ethanol after acetic acid working solution, and with anhydrous ethanol for 3 times. After that, soak in xylene and add drops of neutral gum to seal tablets. Under a high-magnification microscope (Olympus, Japan) the LV collagen content was measured in five visual fields of each section, and four inconsecutive sections from each heart by using Image J software (National Institutes of Health, USA). The interstitial collagen volume fraction (CVF) was calculated as the ratio of the stained collagen area to the total myocardial area.

### Immunofluorescence

Rats were anesthetized with an intraperitoneal injection of urethane (100 mg/kg) and subsequently perfused via the ascending aorta with normal saline followed by 4% paraformaldehyde. Hearts were removed, post-fixed in 4% paraformaldehyde, dehydrated successively in graded sucrose solution at 4 °C, and embedded in OCT. 16 μm slices were cut on a Leica CM1850 cryostat and transferred to silanized slides. For immunolabelling, the sections were washed in PBS for 20 min, and incubated with 0.5% Triton X-100 for 15 min at room temperature. After rinsed three times in PBS, the sections were blocked in 2% BSA for 30 min at room temperature. Then, the sections were incubated with primary anti-mouse IgG (1:500, Santa Cruz, USA) and donkey anti-mouse secondary antibody (1:500, Abcam, UK) conjugated to fluorescein isothiocyanate. After DAPI staining, the sections were cover-slipped with Fluoromont-G mounting medium. The sections (n = 30 from 3 rats) were photographed using a laser scan confocal fluorescent microscope (FV3000, Olympus, Japan).

### Quantitative real-time PCR (qPCR)

Total RNA was extracted from LV free wall tissue using EASYspin Plus Tissue/Cell RNA Rapid Extraction Kit (Beijing Adlai Biological Technology Co., LTD, China) according to the manufacturer’s protocol. qPCR was performed using Two-Step PrimeScript™ RT reagent Kit with gDNA Eraser (Takara, Japan). The sequences of specific primers are listed in a Supplementary File (Table S1). The relative expression levels of targeted mRNAs (RhoA, ROCK) were calculated by the 2^−ΔΔCt^ method, and β-actin was used as an endogenous control.

### Western blot

Protein lysates from LV tissue were prepared and separated by 10% sodium dodecyl sulfate polyacrylamide gel electrophoresis and then transferred onto nitrocellulose membranes (Thermo Fisher Technology Co., LTD, China). The blotted membranes were incubated with primary antibodies against RhoA (1:3000, Santa Cruz, USA), ROCK (Santa Cruz, USA), followed by incubation with goat anti-rabbit IgG (1:3000) was added and incubated at room temperature for 2 h (Abways TECHNOLOGY Inc). The membranes were then developed with chemiluminescence reagents (Shanghai Yase Biotechnology Co., LTD, China). The western blotting signals were analyzed using Tanon-4600sf automatic chemiluminescence image analysis system (Shanghai Tanon Technology Co., LTD, China).

### Statistical analysis

Data are presented as means ± SEM and statistically analyzed using GraphPad Prism 8.4.2 software (GraphPad Software Inc., San Diego, USA). One-way and two-way analysis of variance (ANOVA) followed by the post-hoc Bonferroni test was used for multiple comparisons. P < 0.05 was considered statistically significant.

## Results

### Effect of exercise training on blood pressure

**Figure 2:**
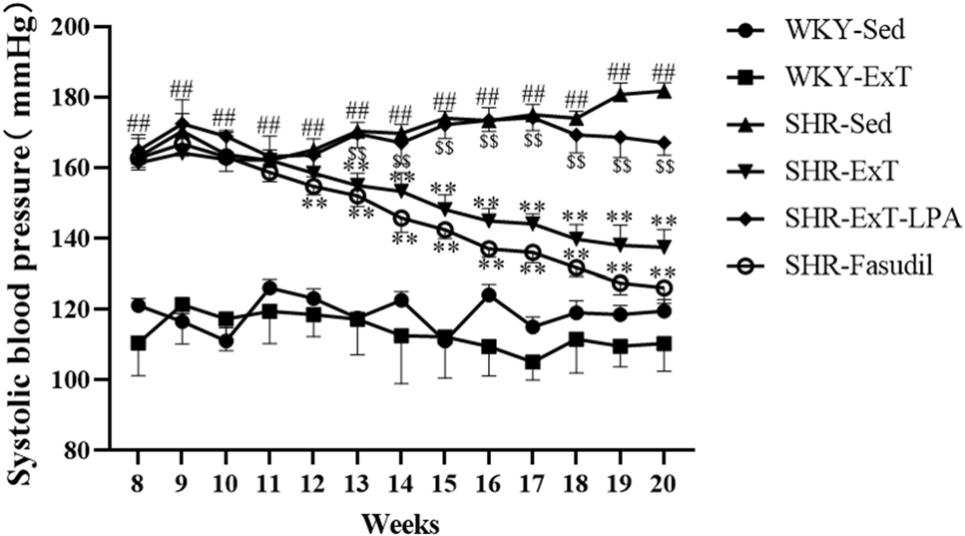
Systolic blood pressure (mmHg) of six groups. *SHR-Sed* SHR control group, *WKY-Sed* WKY control group, *SHR-ExT* SHR exercise group, *WKY-ExT* WKY exercise group, *SHR-ExT-LPA* SHR exercise plus LPA group; *SHR-Fasudil* SHR plus Fasudil group; Data are presented as mean ± SEM. n = 8 in each group. ^#^*P* < 0.05, ^##^*P* < 0.01 *vs* WKY-Sed; ^$^*P* < 0.05, ^$$^*P* < 0.01 *vs* SHR-ExT; *P < 0.05, **P < 0.01 *vs* SHR-Sed.

Compared with WKY rats (age range from 8 to 20 weeks), the systolic blood pressures at all time points were markedly higher in SHR rats (P < 0.01) (Fig. 2). A 12-week swimming training (from 9 to 20 weeks) could significantly decrease the systolic blood pressure of SHR-ExT rats, but it had no effect on blood pressure of WKY-ExT rats. The depressive effect of ExT on SHR blood pressure could be blocked by LPA, an activator of Rho/ROCK signaling pathway (P < 0.01). On the contrary, Fasudil, an inhibitor of the Rho/ROCK signaling pathway could decrease the blood pressure of SHRs (P < 0.01), the depressive effect is consistent with that of ExT (Fig. 2). The results suggest that low-intensity ExT has a depressive effect on the blood pressure of SHRs, which is related with the inhibition of Rho/ROCK signaling pathway.

### Effect of exercise training on thickness of left ventricular wall

**Figure 3:**
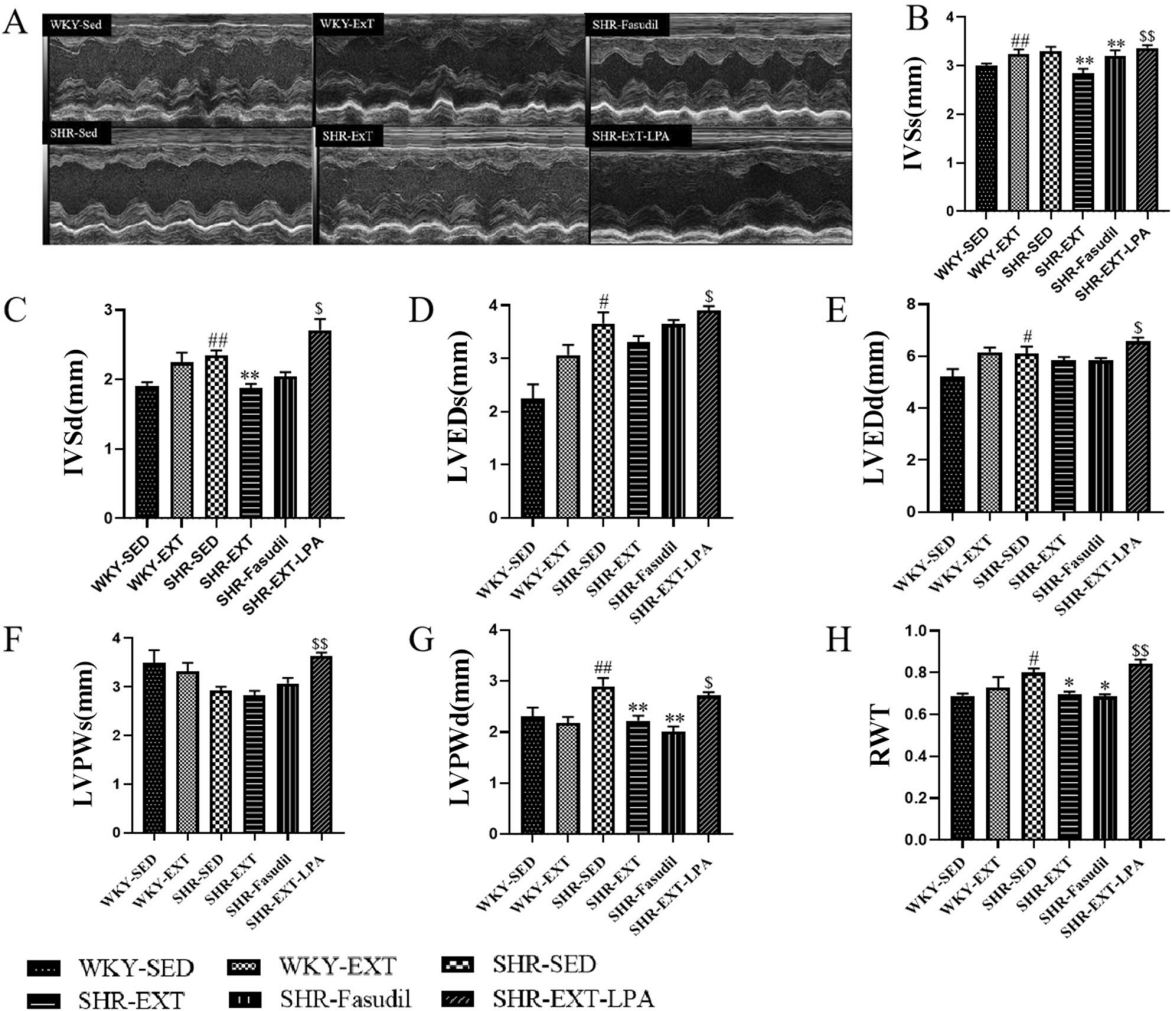
Effect of exercise training on left ventricular function and wall thickness. (**A**) The original ultrasound image. (**B**) IVSs (Systolic interventricular septal); (**C**) IVSd (diastolic interventricular septal); (**D**) LVEDs (left ventricular end-systolic dimension); (**E**) LVEDd (left ventricular end-diastolic dimension); (**F**) LVPWs (left ventricular posterior wall during systole); (**G**) LVPWd (left ventricular posterior wall in diastole); (**H**) RWT (relative wall thickness). *SHR-Sed* SHR control group, *WKY-Sed* WKY control group, *SHR-ExT* SHR exercise group, *WKY-ExT* WKY exercise group, *SHR-ExT-LPA* SHR exercise plus LPA group; *SHR-Fasudil* SHR plus Fasudil group; Data are expressed as mean ± SEM. n = 7 in each group. ^#^*P* < 0.05, ^##^*P* < 0.01 *vs* WKY-Sed; ^$^*P* < 0.05, ^$$^*P* < 0.01 *vs* SHR-ExT; *P < 0.05, **P < 0.01 *vs* SHR-Sed.

**Figure 4:**
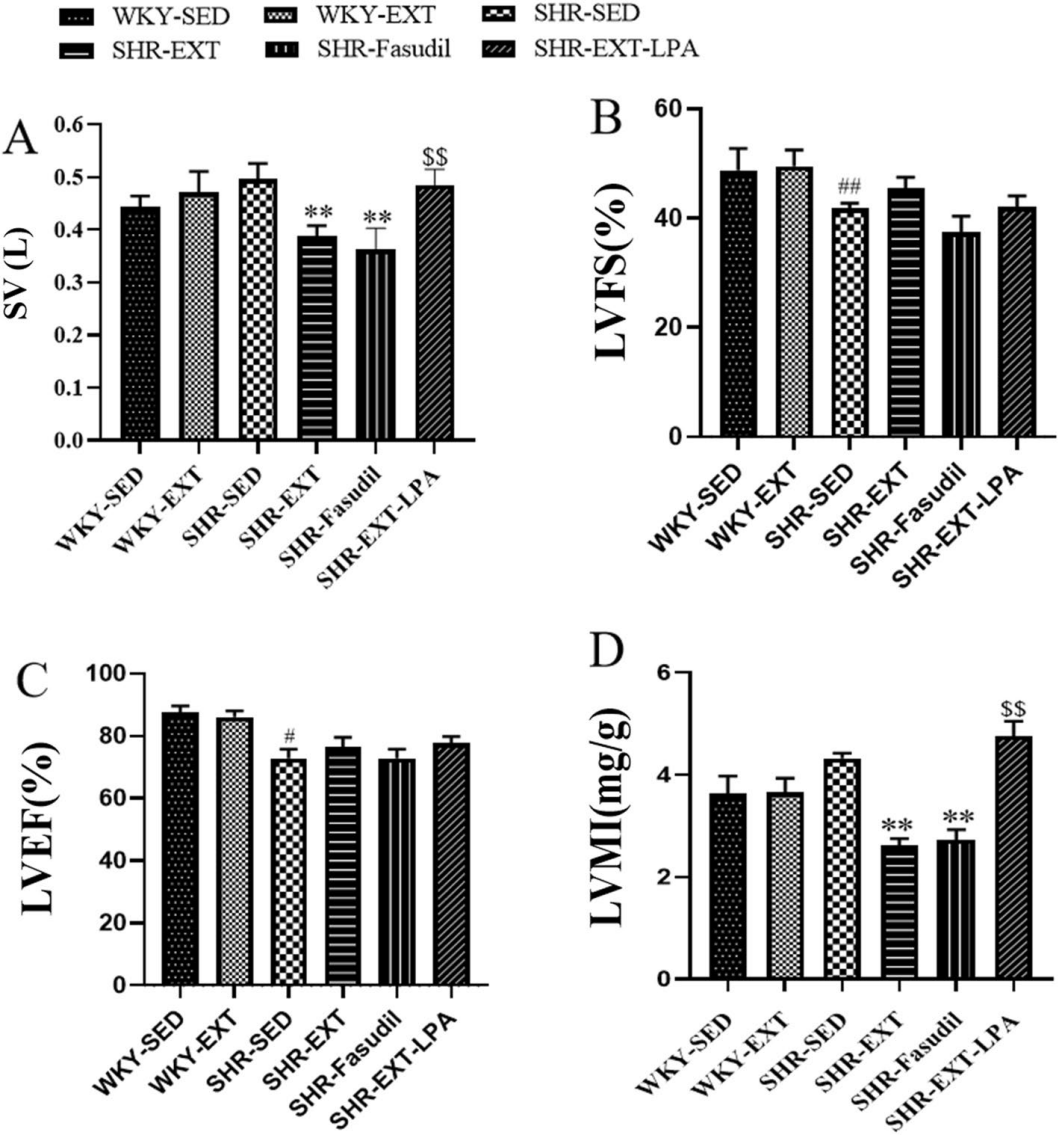
Effect of exercise training on left ventricular function. (**A**) SV (stroke volume); (**B**) FS (fractional shortening); (**C**) EF (ejection fraction), (**D**) LVMI (left ventricular mass index). *SHR-Sed* SHR control group, *WKY-Sed* WKY control group, *SHR-ExT* SHR exercise group, *WKY-ExT* WKY exercise group, *SHR-ExT-LPA* SHR exercise plus LPA group; *SHR-Fasudil* SHR plus Fasudil group; Data are expressed as mean ± SEM. n = 7 in each group. ^#^*P* < 0.05, ^##^*P* < 0.01 *vs* WKY-Sed; ^$^*P* < 0.05, ^$$^*P* < 0.01 *vs* SHR-ExT; *P < 0.05, **P < 0.01 *vs* SHR-Sed.

**Figure 5:**
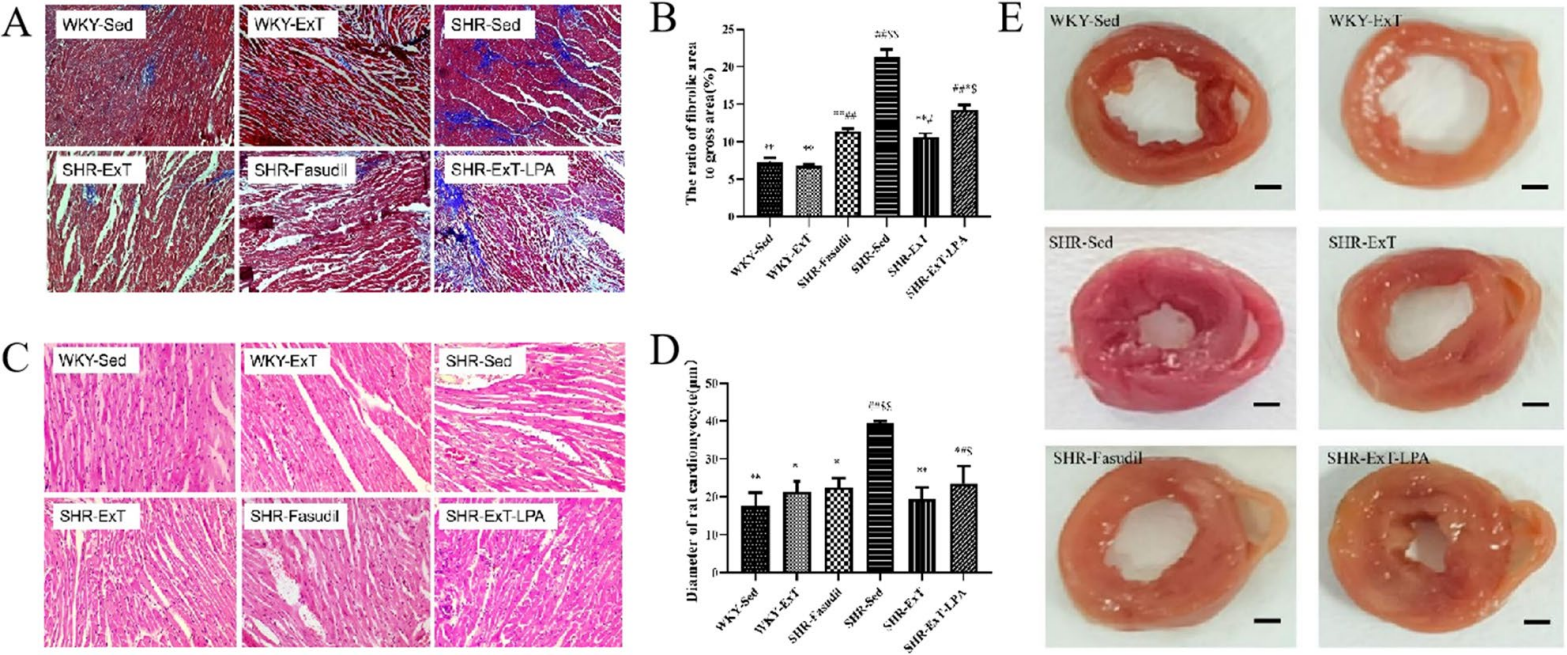
Effect of exercise training on left ventricular structures. (**A,B**) Masson’s trichrome staining, (**C,D**) Hematoxylin staining, (**E**) original graph of the left ventricle. *SHR-Sed* SHR control group, *WKY-Sed* WKY control group, *SHR-ExT* SHR exercise group, *WKY-ExT* WKY exercise group, *SHR-ExT-LPA* SHR exercise plus LPA group; *SHR-Fasudil* SHR plus Fasudil group; Data are expressed as mean ± SEM. n = 3 in each group. ^#^*P* < 0.05, ^##^*P* < 0.01 *vs* WKY-Sed; ^$^*P* < 0.05, ^$$^*P* < 0.01 *vs* SHR-ExT; *P < 0.05, **P < 0.01 *vs* SHR-Sed.

Cardiac ultrasonic data showed that the IVSd, LVEDs, LVEDd and LVPWd were both higher in SHR-Sed compared with those of WKY-Sed rats (P < 0.05 or P < 0.01) (Fig. 3C–E,G). Compared with SHR-Sed rats, IVSs, IVSd, LVPWd and LVMI in SHR-ExT rats were declined significantly (P < 0.01) (Figs. 3B,C,G, 4D). The improving effect of ExT on LVEDd, LVEDs, IVSs, IVSd, LVPWd, LVPWs and LVMI was blocked by LPA in SHR-ExT rats (P < 0.05 or P < 0.01) (Figs. 3B–G, 4D). On the contrary, an improvement effect was induced by Fasudil in SHR rats IVSs, LVMI and LVPWd (P < 0.01) (Figs. 3B,G, 4D). The RWT was significantly larger in SHR-Sed rats and significantly lower in both SHR-ExT and SHR-Fasudil rats than WKY-Sed rats (P < 0.05) (Figs. 3H, 5E). The results suggest that low-intensity ExT has an improved effect on thickness of left ventricular wall in SHR, which is related to the inhibition of Rho/ROCK signaling pathway.

### Effect of exercise training on left ventricular function

The cardiac ultrasonic data showed that the ejection fraction (EF) and left ventricular fractional shortening (FS) rate were decreased in SHR-Sed rats compared with those of WKY-Sed rats (P < 0.05 or P < 0.01) (Fig. 4A–C). There were no statistical significance of LVEF between SHR-Sed and SHR-ExT rats. The SV of SHR-ExT rats was significantly lower than that of the SHR-Sed rats (P < 0.01). The improving effect of ExT on ventricular function in SHR-ExT rats was blocked by LPA (P < 0.01). On the contrary, an improving effect was induced by Fasudil in SHR-Sed rats (P < 0.01) (Figs. 3A, 4A–C). The results suggest that low-intensity ExT has improvement effect on ventricular function in SHR-Sed, which is related with the inhibition of Rho/ROCK signaling pathway.

### Effect of exercise training on myocardial architecture and interstitial collagen

HE staining showed that the structure of cardiomyocytes was disordered, intercellular space and nucleus volume were increased with unclear transverse striation in SHR-Sed rats compared with WKY-Sed rats (P < 0.01). The changes of myocardium were much slighter in SHR-ExT rats than those of SHR-Sed rats (P < 0.01), which indicates the improvement of ExT on myocardial injury of SHR. The improvement of ExT was inhibited by activation of Rho/ROCK signaling pathway (P < 0.05) (Fig. 5C,D).

Masson's trichrome staining showed that interstitial collagen fiber was increased in SHR-Sed rats compared with WKY-Sed rats (P < 0.01), but decreased in SHR-ExT rats contrasted with SHR-Sed rats (P < 0.01), which suggest that ExT has an anti-fibration effect. This anti-fibration effect could be blocked by the activation of Rho/ROCK signaling pathway (P < 0.05) (Fig. 5A,B).

### Effect of exercise training on the expression of RhoA and rock in myocardium

**Figure 6:**
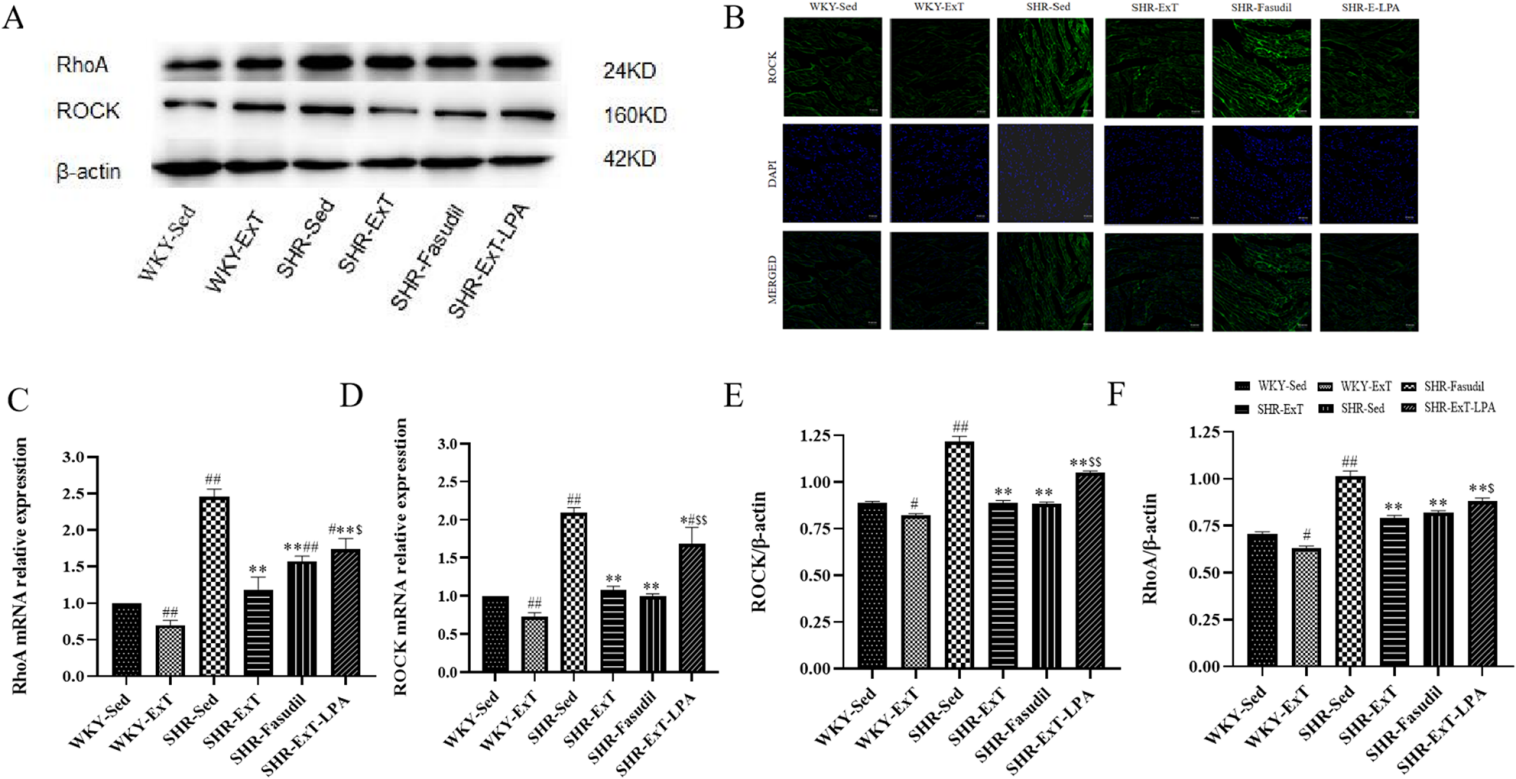
Effect of exercise training on expression of RhoA and ROCK. (**A**) Representative blot images of RhoA and ROCK proteins. (**C,D**) Expression of RhoA and ROCK mRNA. (**E,F**) Expression of RhoA and ROCK proteins. *SHR-Sed* SHR control group, *WKY-Sed* WKY control group, *SHR-ExT* SHR exercise group, *WKY-ExT* WKY exercise group, *SHR-ExT-LPA* SHR exercise plus LPA group; *SHR-Fasudil* SHR plus Fasudil group; data are expressed as mean ± SEM. n = 3 in each group. ^#^*P* < 0.05, ^##^*P* < 0.01 *vs* WKY-Sed; ^$^*P* < 0.05, ^$$^*P* < 0.01 *vs* SHR-ExT; *P < 0.05, **P < 0.01 *vs* SHR-Sed. (**B**) Representative confocal photomicrographs of ROCK-stained and DAPI-stained left ventricular sections. Green fluorescence shows ROCK-positive cells; blue fluorescence shows the nuclei of cardiomyocytes (DAPI-positive). Scale bar = 50 μm.

The expression levels of RhoA and ROCK mRNAs and proteins were up-regulated in SHR-Sed rats compared with WKY-Sed rats (P < 0.01), but they were down-regulated in SHR-ExT rats compared with SHR-Sed rats (P < 0.01). The down-regulation of RhoA and ROCK expression was blocked by LPA in SHR-ExT rats (P < 0.05 or P < 0.01), and the up-regulation of RhoA and ROCK expression was blocked by Fasudil in SHR-Fasudil rats (P < 0.01) (Fig. 6A,C–F).

By immunofluorescent staining, we found that ROCK was abundantly expressed on the cell membrane of left ventricular cardiomyocytes. The intensity of ROCK fluorescence staining was lower in cardiomyocytes of WKY-Sed, WKY-ExT, SHR-ExT, and SHR-Fasudil than SHR-Sed rats. The intensity of ROCK fluorescence staining was significantly enhanced in the SHR-ExT-LPA group compared with SHR-ExT group (Fig. 6B). The results suggest that ExT can depress hypertension through inhibiting Rho/ROCK signaling pathway.

## Discussion

Long-term hypertension can lead to ventricular reactive hypertrophy and remodeling, which is the pathophysiological basis for chronic heart failure^15,16^. The results made in this study showed that the IVSd, LVEDs, LVEDd and LVPWd were increased, the structure of cardiomyocytes was damaged in SHR rats compared with WKY rats, which demonstrated the myocardial remodeling and dysfunction of left ventricular in 20-week-old SHRs. Noteworthily, the left ventricular remodeling and left ventricular diastolic dysfunction in SHR were alleviated by 12-week low-intensity swimming training, which demonstrated the protective effect of ExT against myocardial remodeling and dysfunction induced by hypertension. Furthermore, agonist of Rho/ROCK could counteract the cardiac protective effect of ExT, and antagonist of Rho/ROCK could induce the similar cardiac protection in SHR rats. It suggests that Rho/ROCK plays an important role in the beneficial effect of ExT.

Previous studies have found that the beneficial effect of ExT on cardiovascular system depends on many factors such as training load, duration and ExT frequency^17^. In animals and humans with hypertension, moderate- and low-intensity ExT is considered to be more beneficial to the cardiovascular system than high-intensity ExT^18^. According to the existing literature, the oxygen consumption of exercise animals is 60–70% (68% V_O2m_) of their maximum oxygen consumption in low-intensity ExT^19^. Soares^20^ found that swimming training without weight-bearing (ex 0%) or with weight-bearing 3% of self-weight (ex 3%) had beneficial effects on hypertension, high heart rate and cardiac dysfunction in 2K1C rats. Ex 0% was better for improving the cardiac changes in renovascular hypertensive rats. Ex 5% exercise was often thought of as high-intensity ExT would lead to adverse effects^18^. With reference to the scheme of other laboratory^19^. Low-intensity ExT was used to explore the mechanism underlying the improving effect of ExT on cardiac remodeling in SHR.

It is well known that the heart is one of the main target organs damaged by high blood pressure^21^. Under hypertension condition, the afterload of heart increases and myocardial contractility must be enhanced in order to maintain adequate cardiac output. In the case of long-term overload, the myocardium undergoes a variety of morphological and functional changes, in which the most significant morphological structural damage is hypertrophy and myocardial remodeling. The left ventricular hypertrophy can cause significant myocardial contraction and diastolic dysfunction, as well as myocardial ischemia duo to decreased coronary flow, and even serious events such as arrhythmias, heart failure, and myocardial infarction^22^. Therefore, effectively lowering arterial blood pressure is an important measure to prevent or treat hypertensive cardiac damage in hypertension^23^. Consistent with previous studies that ExT has hypotensive effect^24^, our result showed ExT decreased the arterial blood pressure through inhibiting Rho/ROCK pathway in SHR. It suggests that the antihypertensive effect of ExT might be one of mechanisms for the cardiac protective effect against structure and function damage in SHR.

RhoA/ROCK signaling pathway is considered to be a major regulator of vascular tone and arterial blood pressure, and the overactivation of this pathway is involved in the pathogenesis of hypertension^10^. RhoA and ROCK proteins of the RhoA/ROCK signal pathway appear a high level of expression and activity in spontaneously hypertensive rats and patients^8^. The down-regulation of ROCK expression can reduce cardiac fibrosis^11^, and the specific deletion of ROCK2 in fibroblasts can alleviate cardiac hypertrophy, fibrosis and diastolic dysfunction through inhibiting the production of fibroblast growth factor FGF^12^. In this study, we found that the expression of RhoA and ROCK mRNAs and proteins was up-regulated in SHR rats, and ExT down-regulated the expression of RhoA and ROCK mRNAs and proteins in SHR. Also the beneficial effects of ExT on high blood pressure and cardiac damage were abolished by inhibition of RhoA/ROCK signal pathway and induced by activation of RhoA/ROCK signal pathway in SHR. It suggests that the cardiac damage in SHR is related with overexpression of RhoA/ROCK signaling, and protective effect of ExT on heart is carried out through inhibiting RhoA/ROCK signaling.

In conclusion, low-intensity ExT can reduce the blood pressure and improve cardiac architecture and functional damage through inhibiting RhoA/ROCK signaling pathway in SHR, which provides a new idea or target for the prevention and treatment of hypertension and hypertensive heart injury.

## Supplementary Information

Supplementary Table S1.

Supplementary Information 1.

Supplementary Information 2.

Supplementary Figures.

## Data Availability

The datasets generated during and/or analyzed during the current study are available from the corresponding author on reasonable request.
