# Supplementary material for: Inhibition of Rho/ROCK signaling pathway participates in the cardiac protection of exercise training in spontaneously hypertensive rats
Source: Sci Rep. 2022 Oct 25;12:17903. doi: 10.1038/s41598-022-22191-3 (PMC9596711; doi:10.1038/s41598-022-22191-3)
Supplement: Supplementary file 1 — Supplementary Table S1. [file 41598_2022_22191_MOESM1_ESM.docx]

**Supplementary**

Table S1. The primers used for quantitative PCR.

| Gene | Forward Primer | Reverse Primer |
| --- | --- | --- |
| ROCK | 5’ACAGGGAGGTACGACTTGGAAG3’ | 5’ACCACTGGAGCTGCCGTCTC3’ |
| RhoA | 5’AGCAAGGACCAGTTCCCAGAGG3’ | 5’TCTACCTGCTTCCCGTCCACTTC3’ |
| β-Tubulin | 5’CAACGCCACGCTGTCCATCC3’ | 5’GCGGAAGCAGATGTCGTAGAGC3’ |
