# Supplementary material for: Inhibition of Rho/ROCK signaling pathway participates in the cardiac protection of exercise training in spontaneously hypertensive rats
Source: Sci Rep. 2022 Oct 25;12:17903. doi: 10.1038/s41598-022-22191-3 (PMC9596711; doi:10.1038/s41598-022-22191-3)

Supplementary: The Western Blot raw images of RhoA and ROCK

**Figure S1** The raw image of β-actin staining.


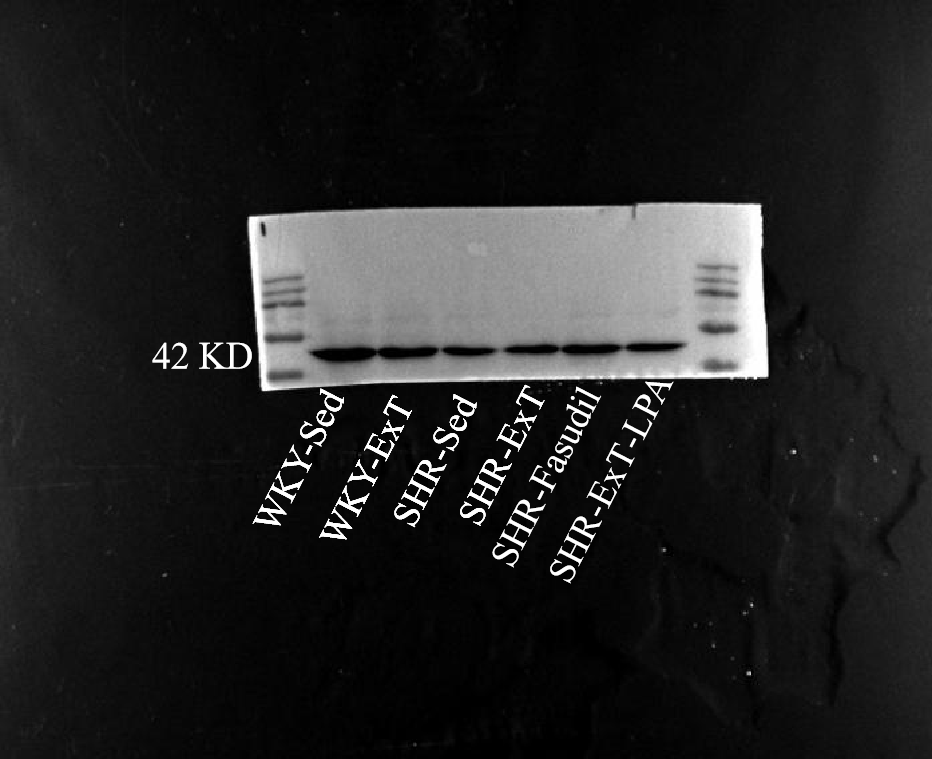


**Figure S2** The raw image of RhoA staining.


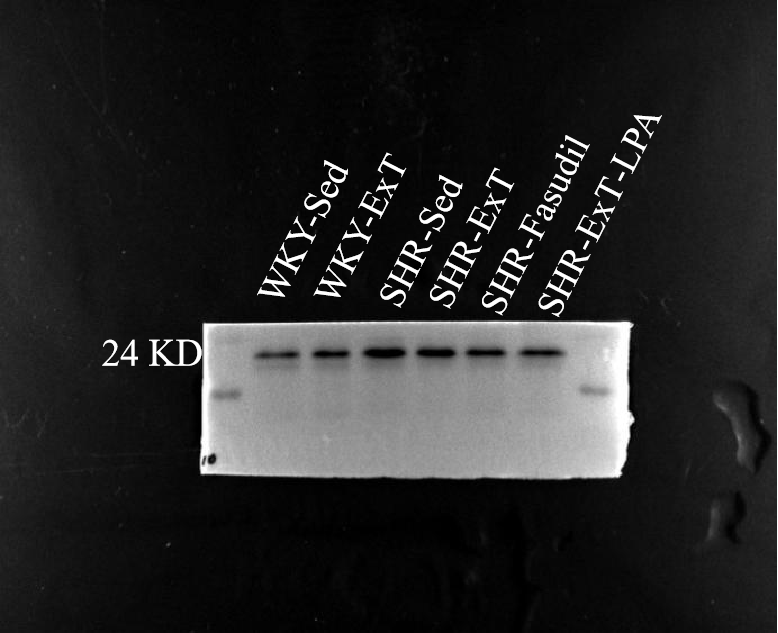


**Figure S3** The raw image of ROCK staining.


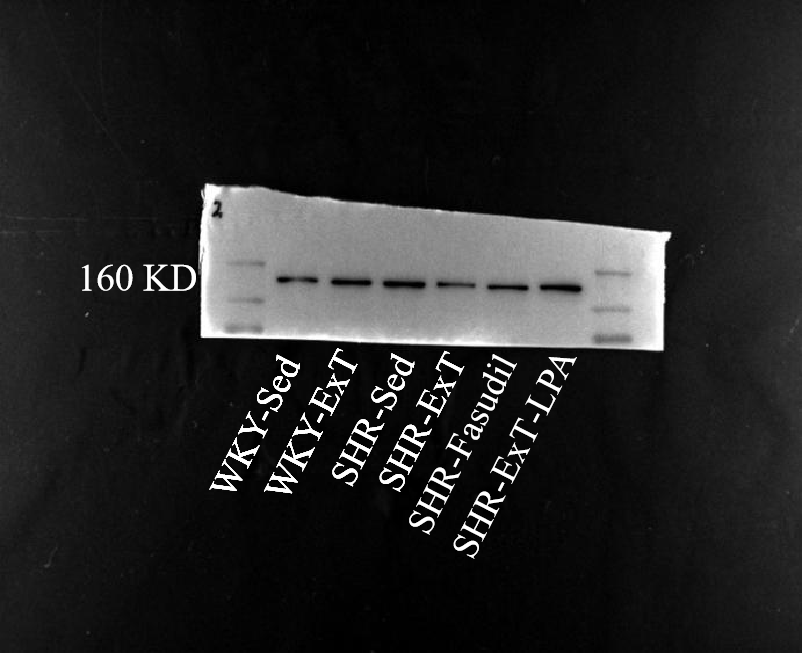

Supplement: Supplementary file 4 — Supplementary Figures. [file 41598_2022_22191_MOESM4_ESM.docx]
